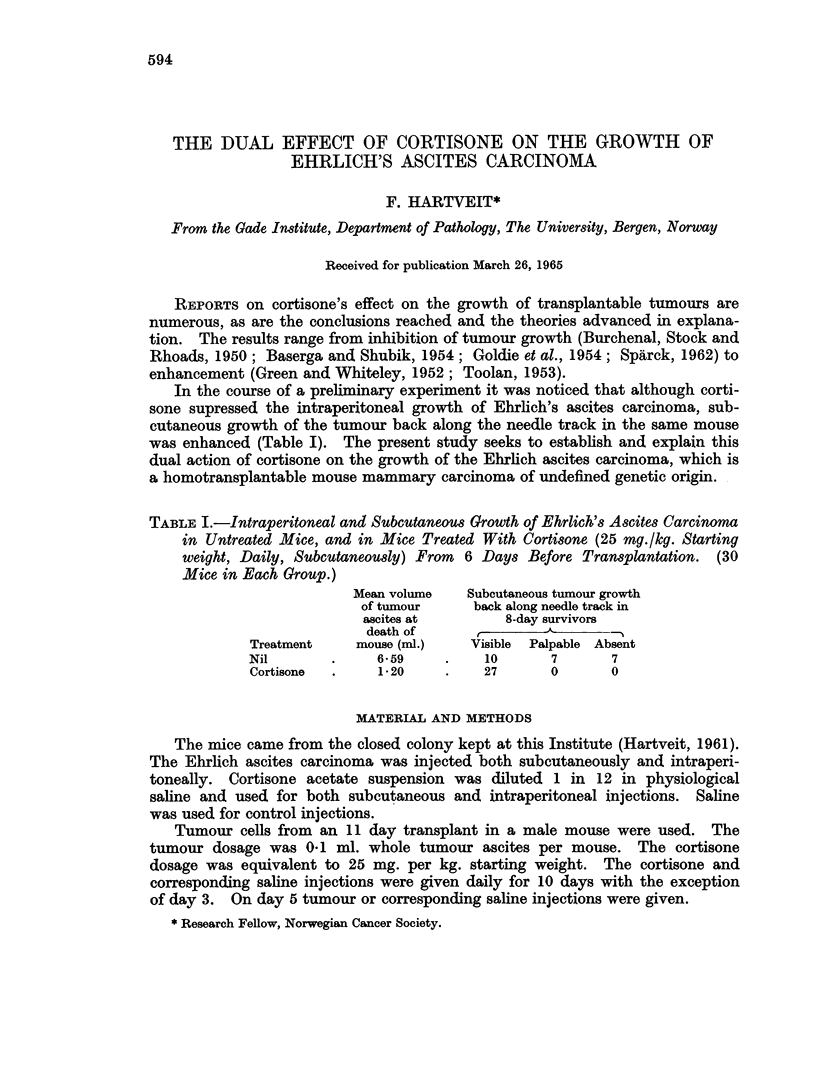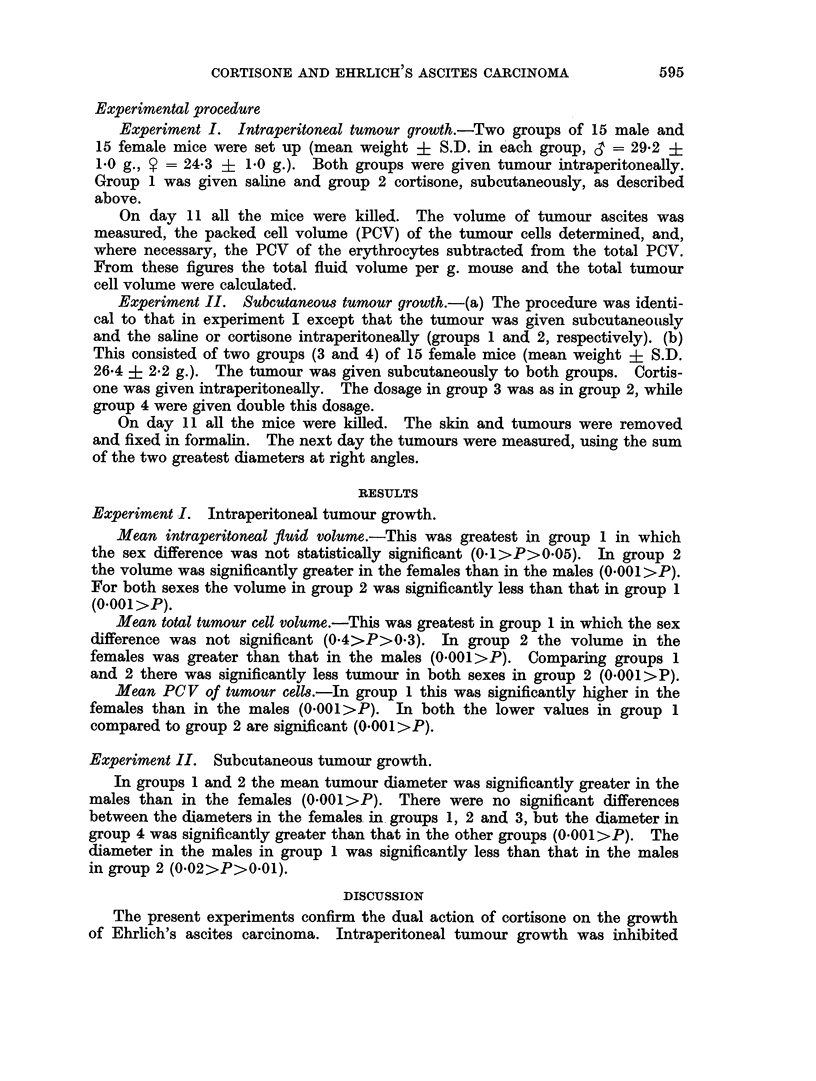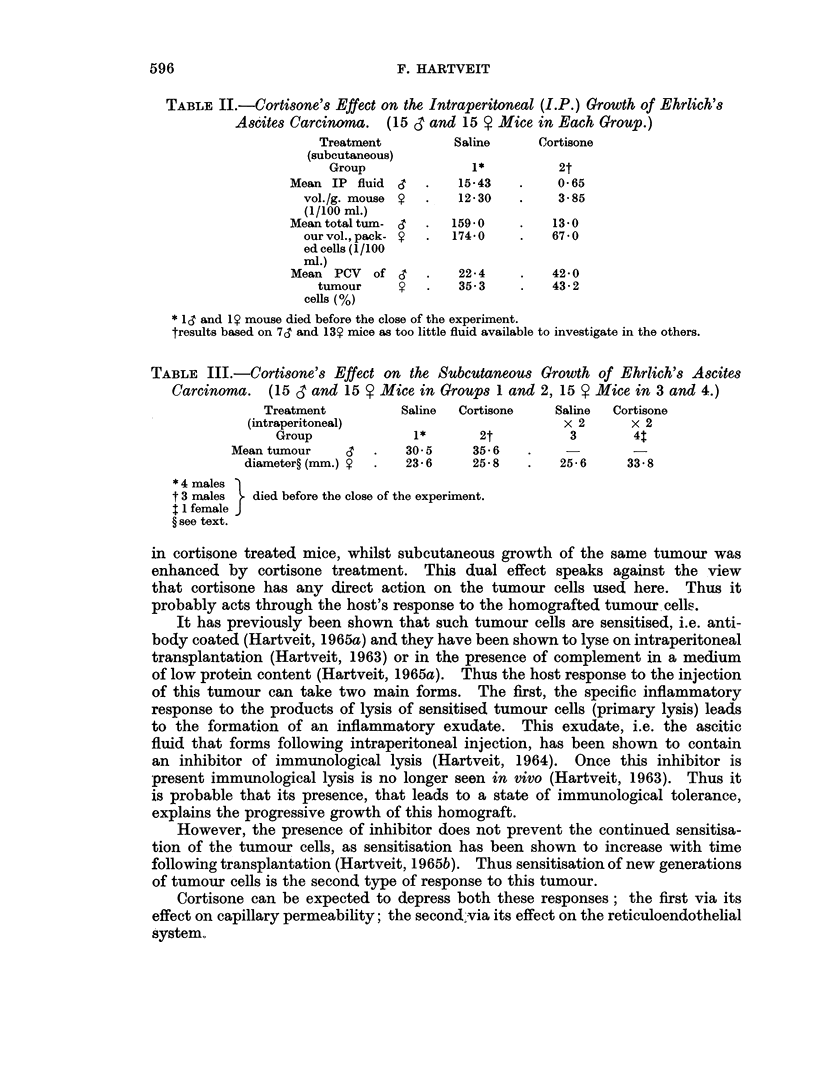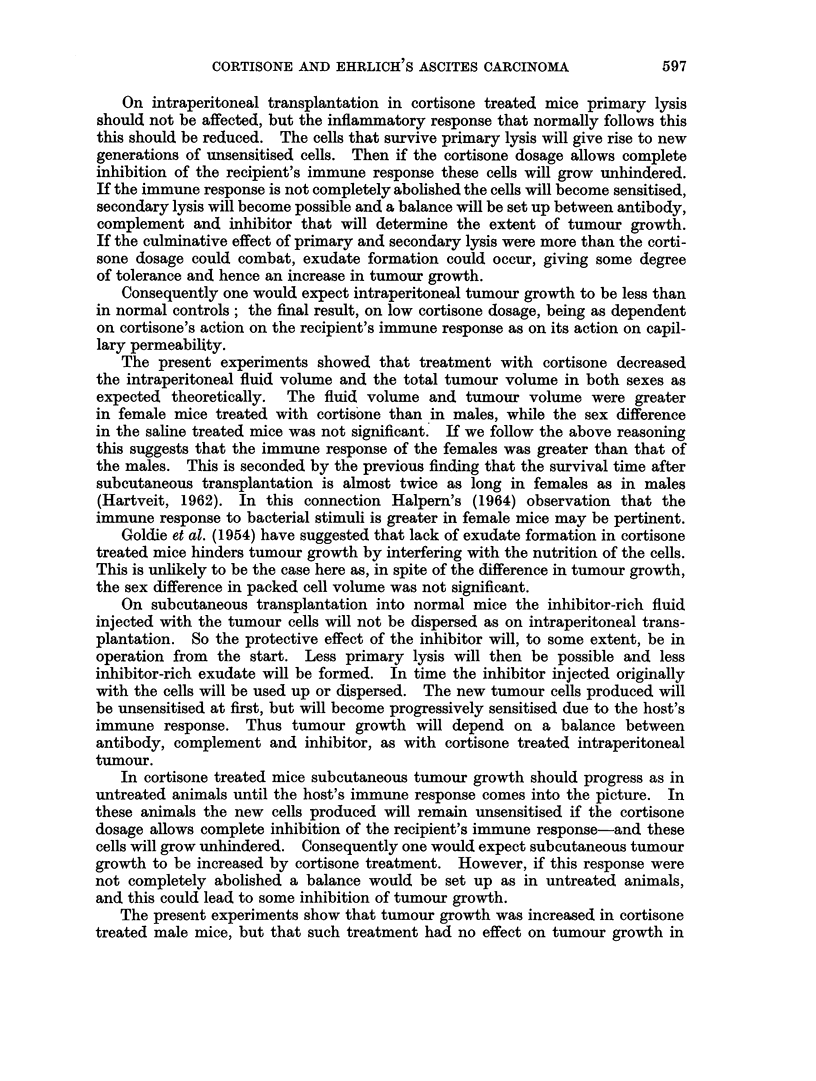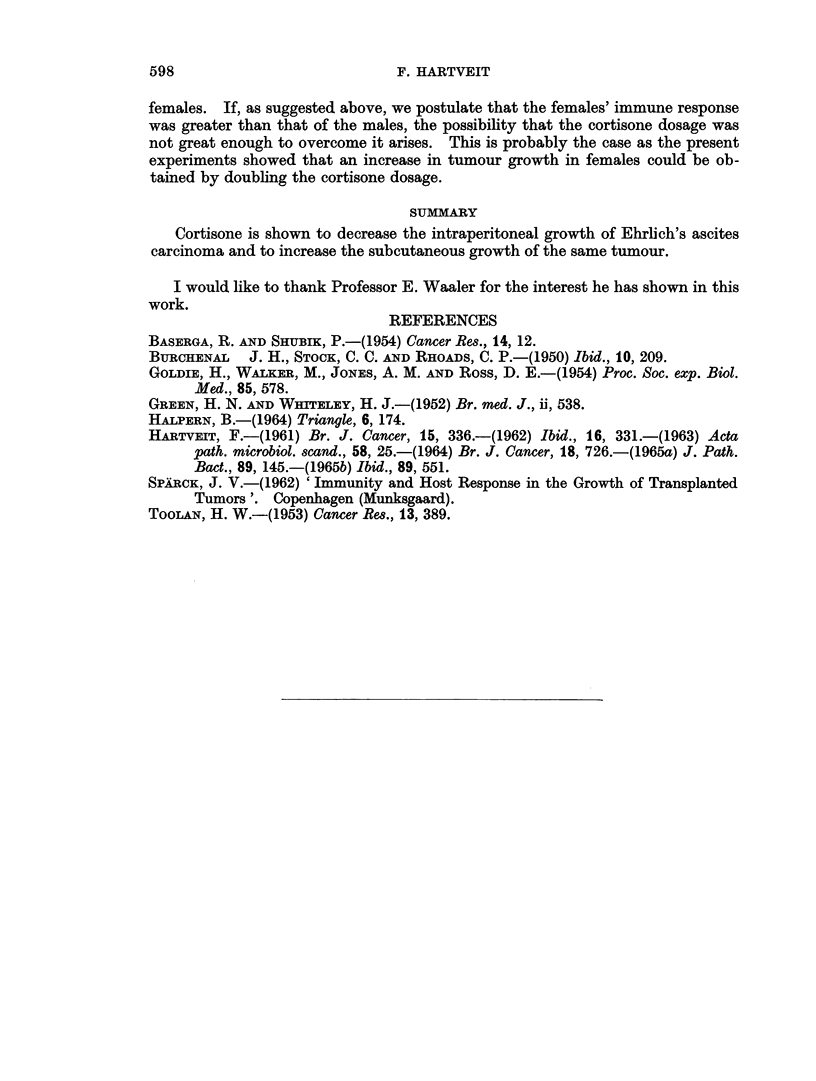# The dual effect of cortisone on the growth of Ehrlich's ascites carcinoma.

**DOI:** 10.1038/bjc.1965.74

**Published:** 1965-09

**Authors:** F. Hartveit


### THE DUAL EFFECT OF CORTISONE ON THE GROWTH OF EHRLICH'S ASCITES CARCINOMA

F. HARTVEIT*

*From the Gade Institute, Department of Pathology, The University, Bergen, Norway*

Received for publication March 26, 1965

REPORTS on cortisone's effect on the growth of transplantable tumours are numerous, as are the conclusions reached and the theories advanced in explanation. The results range from inhibition of tumour growth (Burchenal, Stock and Rhoads, 1950 ; Baserga and Shubik, 1954 ; Goldie *et al.*, 1954 ; Spärck, 1962) to enhancement (Green and Whiteley, 1952 ; Toolan, 1953).

In the course of a preliminary experiment it was noticed that although cortisone supressed the intraperitoneal growth of Ehrlich's ascites carcinoma, subcutaneous growth of the tumour back along the needle track in the same mouse was enhanced (Table I). The present study seeks to establish and explain this dual action of cortisone on the growth of the Ehrlich ascites carcinoma, which is a homotransplantable mouse mammary carcinoma of undefined genetic origin.

TABLE I.—*Intraperitoneal and Subcutaneous Growth of Ehrlich's Ascites Carcinoma in Untreated Mice, and in Mice Treated With Cortisone* (25 *mg./kg. Starting weight, Daily, Subcutaneously*) *From* 6 *Days Before Transplantation.* (30 *Mice in Each Group.*)

| Treatment | Mean volume of tumour ascites at death of mouse (ml.) | Subcutaneous tumour growth back along needle track in 8-day survivors | | |
|---|---|---|---|---|
| | | Visible | Palpable | Absent |
| Nil | 6·59 | 10 | 7 | 7 |
| Cortisone | 1·20 | 27 | 0 | 0 |

### MATERIAL AND METHODS

The mice came from the closed colony kept at this Institute (Hartveit, 1961). The Ehrlich ascites carcinoma was injected both subcutaneously and intraperitoneally. Cortisone acetate suspension was diluted 1 in 12 in physiological saline and used for both subcutaneous and intraperitoneal injections. Saline was used for control injections.

Tumour cells from an 11 day transplant in a male mouse were used. The tumour dosage was 0·1 ml. whole tumour ascites per mouse. The cortisone dosage was equivalent to 25 mg. per kg. starting weight. The cortisone and corresponding saline injections were given daily for 10 days with the exception of day 3. On day 5 tumour or corresponding saline injections were given.

* Research Fellow, Norwegian Cancer Society.

*Experimental procedure*

*Experiment I. Intraperitoneal tumour growth.*—Two groups of 15 male and 15 female mice were set up (mean weight $\pm$ S.D. in each group, ♂ = 29·2 $\pm$ 1·0 g., ♀ = 24·3 $\pm$ 1·0 g.). Both groups were given tumour intraperitoneally. Group 1 was given saline and group 2 cortisone, subcutaneously, as described above.

On day 11 all the mice were killed. The volume of tumour ascites was measured, the packed cell volume (PCV) of the tumour cells determined, and, where necessary, the PCV of the erythrocytes subtracted from the total PCV. From these figures the total fluid volume per g. mouse and the total tumour cell volume were calculated.

*Experiment II. Subcutaneous tumour growth.*—(a) The procedure was identical to that in experiment I except that the tumour was given subcutaneously and the saline or cortisone intraperitoneally (groups 1 and 2, respectively). (b) This consisted of two groups (3 and 4) of 15 female mice (mean weight $\pm$ S.D. 26·4 $\pm$ 2·2 g.). The tumour was given subcutaneously to both groups. Cortisone was given intraperitoneally. The dosage in group 3 was as in group 2, while group 4 were given double this dosage.

On day 11 all the mice were killed. The skin and tumours were removed and fixed in formalin. The next day the tumours were measured, using the sum of the two greatest diameters at right angles.

### RESULTS

*Experiment I.* Intraperitoneal tumour growth.

*Mean intraperitoneal fluid volume.*—This was greatest in group 1 in which the sex difference was not statistically significant ($0{\cdot}1 > P > 0{\cdot}05$). In group 2 the volume was significantly greater in the females than in the males ($0{\cdot}001 > P$). For both sexes the volume in group 2 was significantly less than that in group 1 ($0{\cdot}001 > P$).

*Mean total tumour cell volume.*—This was greatest in group 1 in which the sex difference was not significant ($0{\cdot}4 > P > 0{\cdot}3$). In group 2 the volume in the females was greater than that in the males ($0{\cdot}001 > P$). Comparing groups 1 and 2 there was significantly less tumour in both sexes in group 2 ($0{\cdot}001 > \mathrm{P}$).

*Mean PCV of tumour cells.*—In group 1 this was significantly higher in the females than in the males ($0{\cdot}001 > P$). In both the lower values in group 1 compared to group 2 are significant ($0{\cdot}001 > P$).

*Experiment II.* Subcutaneous tumour growth.

In groups 1 and 2 the mean tumour diameter was significantly greater in the males than in the females ($0{\cdot}001 > P$). There were no significant differences between the diameters in the females in groups 1, 2 and 3, but the diameter in group 4 was significantly greater than that in the other groups ($0{\cdot}001 > P$). The diameter in the males in group 1 was significantly less than that in the males in group 2 ($0{\cdot}02 > P > 0{\cdot}01$).

### DISCUSSION

The present experiments confirm the dual action of cortisone on the growth of Ehrlich's ascites carcinoma. Intraperitoneal tumour growth was inhibited

TABLE II.—*Cortisone's Effect on the Intraperitoneal (I.P.) Growth of Ehrlich's Ascites Carcinoma.* (15 ♂ *and* 15 ♀ *Mice in Each Group.*)

| Treatment (subcutaneous) | | Saline | Cortisone |
|---|---|---|---|
| Group | | 1* | 2† |
| Mean IP fluid vol./g. mouse (1/100 ml.) | ♂ | 15·43 | 0·65 |
| | ♀ | 12·30 | 3·85 |
| Mean total tumour vol., packed cells (1/100 ml.) | ♂ | 159·0 | 13·0 |
| | ♀ | 174·0 | 67·0 |
| Mean PCV of tumour cells (%) | ♂ | 22·4 | 42·0 |
| | ♀ | 35·3 | 43·2 |

* 1♂ and 1♀ mouse died before the close of the experiment.
†results based on 7♂ and 13♀ mice as too little fluid available to investigate in the others.

TABLE III.—*Cortisone's Effect on the Subcutaneous Growth of Ehrlich's Ascites Carcinoma.* (15 ♂ *and* 15 ♀ *Mice in Groups* 1 *and* 2, 15 ♀ *Mice in* 3 *and* 4.)

| Treatment (intraperitoneal) | | Saline | Cortisone | Saline × 2 | Cortisone × 2 |
|---|---|---|---|---|---|
| Group | | 1* | 2† | 3 | 4‡ |
| Mean tumour diameter§ (mm.) | ♂ | 30·5 | 35·6 | — | — |
| | ♀ | 23·6 | 25·8 | 25·6 | 33·8 |

* 4 males, † 3 males, ‡ 1 female: died before the close of the experiment.

§ see text.

in cortisone treated mice, whilst subcutaneous growth of the same tumour was enhanced by cortisone treatment. This dual effect speaks against the view that cortisone has any direct action on the tumour cells used here. Thus it probably acts through the host's response to the homografted tumour cells.

It has previously been shown that such tumour cells are sensitised, i.e. antibody coated (Hartveit, 1965*a*) and they have been shown to lyse on intraperitoneal transplantation (Hartveit, 1963) or in the presence of complement in a medium of low protein content (Hartveit, 1965*a*). Thus the host response to the injection of this tumour can take two main forms. The first, the specific inflammatory response to the products of lysis of sensitised tumour cells (primary lysis) leads to the formation of an inflammatory exudate. This exudate, i.e. the ascitic fluid that forms following intraperitoneal injection, has been shown to contain an inhibitor of immunological lysis (Hartveit, 1964). Once this inhibitor is present immunological lysis is no longer seen *in vivo* (Hartveit, 1963). Thus it is probable that its presence, that leads to a state of immunological tolerance, explains the progressive growth of this homograft.

However, the presence of inhibitor does not prevent the continued sensitisation of the tumour cells, as sensitisation has been shown to increase with time following transplantation (Hartveit, 1965*b*). Thus sensitisation of new generations of tumour cells is the second type of response to this tumour.

Cortisone can be expected to depress both these responses ; the first via its effect on capillary permeability ; the second via its effect on the reticuloendothelial system.

On intraperitoneal transplantation in cortisone treated mice primary lysis should not be affected, but the inflammatory response that normally follows this this should be reduced. The cells that survive primary lysis will give rise to new generations of unsensitised cells. Then if the cortisone dosage allows complete inhibition of the recipient's immune response these cells will grow unhindered. If the immune response is not completely abolished the cells will become sensitised, secondary lysis will become possible and a balance will be set up between antibody, complement and inhibitor that will determine the extent of tumour growth. If the culminative effect of primary and secondary lysis were more than the cortisone dosage could combat, exudate formation could occur, giving some degree of tolerance and hence an increase in tumour growth.

Consequently one would expect intraperitoneal tumour growth to be less than in normal controls ; the final result, on low cortisone dosage, being as dependent on cortisone's action on the recipient's immune response as on its action on capillary permeability.

The present experiments showed that treatment with cortisone decreased the intraperitoneal fluid volume and the total tumour volume in both sexes as expected theoretically. The fluid volume and tumour volume were greater in female mice treated with cortisone than in males, while the sex difference in the saline treated mice was not significant. If we follow the above reasoning this suggests that the immune response of the females was greater than that of the males. This is seconded by the previous finding that the survival time after subcutaneous transplantation is almost twice as long in females as in males (Hartveit, 1962). In this connection Halpern's (1964) observation that the immune response to bacterial stimuli is greater in female mice may be pertinent.

Goldie *et al.* (1954) have suggested that lack of exudate formation in cortisone treated mice hinders tumour growth by interfering with the nutrition of the cells. This is unlikely to be the case here as, in spite of the difference in tumour growth, the sex difference in packed cell volume was not significant.

On subcutaneous transplantation into normal mice the inhibitor-rich fluid injected with the tumour cells will not be dispersed as on intraperitoneal transplantation. So the protective effect of the inhibitor will, to some extent, be in operation from the start. Less primary lysis will then be possible and less inhibitor-rich exudate will be formed. In time the inhibitor injected originally with the cells will be used up or dispersed. The new tumour cells produced will be unsensitised at first, but will become progressively sensitised due to the host's immune response. Thus tumour growth will depend on a balance between antibody, complement and inhibitor, as with cortisone treated intraperitoneal tumour.

In cortisone treated mice subcutaneous tumour growth should progress as in untreated animals until the host's immune response comes into the picture. In these animals the new cells produced will remain unsensitised if the cortisone dosage allows complete inhibition of the recipient's immune response—and these cells will grow unhindered. Consequently one would expect subcutaneous tumour growth to be increased by cortisone treatment. However, if this response were not completely abolished a balance would be set up as in untreated animals, and this could lead to some inhibition of tumour growth.

The present experiments show that tumour growth was increased in cortisone treated male mice, but that such treatment had no effect on tumour growth in females. If, as suggested above, we postulate that the females' immune response was greater than that of the males, the possibility that the cortisone dosage was not great enough to overcome it arises. This is probably the case as the present experiments showed that an increase in tumour growth in females could be obtained by doubling the cortisone dosage.

### SUMMARY

Cortisone is shown to decrease the intraperitoneal growth of Ehrlich's ascites carcinoma and to increase the subcutaneous growth of the same tumour.

I would like to thank Professor E. Waaler for the interest he has shown in this work.

### REFERENCES

Baserga, R. and Shubik, P.—(1954) *Cancer Res.*, **14**, 12.
Burchenal J. H., Stock, C. C. and Rhoads, C. P.—(1950) *Ibid.*, **10**, 209.
Goldie, H., Walker, M., Jones, A. M. and Ross, D. E.—(1954) *Proc. Soc. exp. Biol. Med.*, **85**, 578.
Green, H. N. and Whiteley, H. J.—(1952) *Br. med. J.*, ii, 538.
Halpern, B.—(1964) *Triangle*, **6**, 174.
Hartveit, F.—(1961) *Br. J. Cancer*, **15**, 336.—(1962) *Ibid.*, **16**, 331.—(1963) *Acta path. microbiol. scand.*, **58**, 25.—(1964) *Br. J. Cancer*, **18**, 726.—(1965*a*) *J. Path. Bact.*, **89**, 145.—(1965*b*) *Ibid.*, **89**, 551.
Spärck, J. V.—(1962) ' Immunity and Host Response in the Growth of Transplanted Tumors '. Copenhagen (Munksgaard).
Toolan, H. W.—(1953) *Cancer Res.*, **13**, 389.